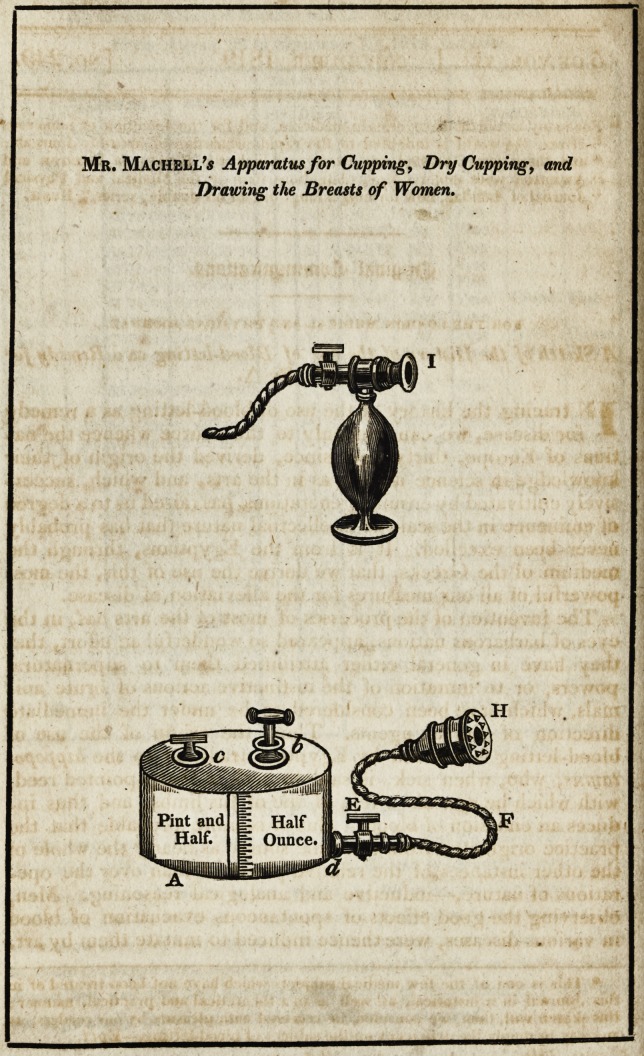# Description of an Apparatus for Cupping, Dry Cupping, and Drawing Milk from the Breasts of Females; with Some Observations Respecting Its Use

**Published:** 1819-11

**Authors:** Thomas Machell

**Affiliations:** Member of the Royal College of Surgeons in London.


FOR THE LONDON MEDICAL AND PHYSICAL JOURNAL.

### *Description of an Apparatus for Cupping, Dry Cupping, and Drawing Milk from the Breasts of Females; with some Observations respecting its Use.* By Mr. THOMAS MACHELL, Member of the Royal College of Surgeons in London.

[WITH AN ENGRAVING.]

I TRANSMIT the following description of my patent apparatus for Cupping, &c. and observations on the mode of using it, for insertion in the Medical Journal.

The body of the apparatus, A, is of an oval form, having two orifices on the top, *b* and *c*. Into *b* is screwed an exhausting syringe or pump, and into *c* a screw-plug, for the purpose of opening and closing that orifice.

At one end of the body of the apparatus is fitted a stop-cock, E, to which is attached the flexible pipe, F; at the extremity of which, the different apparatus for cupping or drawing milk, as hereafter described, is to be affixed.

---

**Mr. Machell's** *Apparatus for Cupping, Dry Cupping, and Drawing the Breasts of Women.*

In order to perform the operation of dry-cupping, the glass, H, is to be screwed to the end of the flexible pipe, F; and an exhaustion must be made in the body of the instrument, by making about thirty strokes of the pump, and by applying the mouth of the glass, H, to that part of the body of the patient to be operated upon; and, by turning the stop-cock more or less, the skin will be raised as much as is required; and the glass may be instantaneously removed, by unscrewing the plug, C, and letting-in the air.

For the purpose of cupping, scarifying, and abstracting blood, at one operation, connected with the extremity of the flexible tube passing from the exhausting-box, is a glass or metal bell, resembling in great measure the common syringe cupping-glass, into which is adjusted a simple piece of mechanism, whereby a plate, on which is fixed the lancet-points, after puncturing the elevated integuments, is disengaged from a catch, by pressure of the soft parts rising into the partially-exhausted vessel, on the button of a delicate spring, by the previous adjustment of which, the extent of the punctures or incisions may be very accurately regulated.

The lancet-bell, after exhausting, by about forty strokes of the piston, the body of the apparatus, is applied to the part from which it is intended to draw blood; and, by turning the stop-cock in the connecting tube, communication is made betwixt the two vessels; the integuments rise into the bell, press against, and are wounded by, the lancets or prickers, disengaged at an accurately-determined moment, and the blood is drawn from the orifices into the exhausted receiver. A cock in the exhausting-box, by admitting at any time the excluded atmosphere, removes the pressure, and liberates the apparatus.

The transparent crystal, which is let into the side of the exhausting-box, admits of a clear view of the blood, or other fluid, flowing into the instrument. By the side of the crystal, and upon the exhausting-box, is a scale; the division of which begins at half-an-ounce, and, in the ordinary-sized apparatus, is continued to the degree of a pint and half: the precise quantity of blood can thereby be measured with much accuracy.

The important advantages gained in the use of this apparatus,—of facility, precision, simplicity, neatness,—are incalculably surpassed by the power of its application to any part whatever of the surface, under any circumstances indicating its propriety, and by any person untrained to the manual dexterity of a professed cupper; and even without inspection of the part to which it is applied,—a circumstance frequently of much importance in female patients. The bone, or its periosteum, covered only by extenuated integument, can never be injured; the skin and its vessels simply are divided, and that to any nicely-determined object, and into any desired point. There is no alarming preparation, no harassing change of apparatus, no exposure. Cleanliness, decency, and the quiet and mental tranquillity of the patient, are in no way infringed upon.

The delicate covering of the hydrocephalic infant's cranium may be depleted, without danger, without alarm, without trouble or delay.

In drawing the breasts, about four strokes of the pump will in general be found sufficient; and the nipple-glass, I, being screwed upon the end of the flexible pipe, the mouth of the glass must be applied to the breast; when the suction may be regulated according to the feelings of the party using the apparatus, by merely turning the handle of the stop-cock more or less; and, in order to remove the glass from the breast without difficulty, it is only necessary to unscrew the plug, when it will become detached.

The annoying, and often in their consequences seriously injurious, difficulties of abstracting milk from the imperfectly-developed nipple of young mothers, are too familiar to the practitioner in the department of midwifery, to need, in this place, more than simple mention. The inadequate and clumsy contrivance hitherto employed, gives place to the convenience, the precision, and the sufficiency, of this almost self-acting apparatus.

The delicate or exhausted female has but to apply the nipple-glass, without being disturbed even from a recumbent position, and regulate, simply by turning the stop-cock, the draught on her breast to the extent which her own feelings dictate as sufficient. The breast which, from ulcerated or excoriated nipple, cannot be emptied of its fluid by suction of the infant, but with almost insufferable agony to the mother, can, by this contrivance, be drawn without pain, and without the perpetual renewed irritation to the ulcerated part, which is the exclusive impediment to the process of healing.

By the adoption of a glass receiver to the neck of the nipple-cap, and which is detached or affixed by a screw-neck, the milk is received uncontaminated, and appropriable to the nutrition of the infant.

*Great Ryder-street, St. James's;*
*Oct.* 8, 1819.

## Figures and Tables

**Figure f1:**